# About Twenty-five Books Reviewed or Noticed

**Published:** 1891-05

**Authors:** 


### Book Notices.

J. B. LIPPINCOTT COMPANY will, beginning with April, issue quarterly thereafter a work entitled "International Clinics." This work will comprise the best and most practical clinical lectures on medicine, surgery, gynecology, pediatrics, dermatology, laryngology, ophthalmology and otology, delivered in the leading medical colleges of this country, Great Britain and Canada. These lectures have been reported by competent medical stenographers, and thoroughly revised by the professors and lecturers themselves. The object of the work is to furnish the busy practitioner and medical student with the most practical clinical instruction, in concise form. Each volume will consist of over 350 octavo pages, illustrated with photographic reproductions of important cases.

A COMPEND OF DISEASES OF CHILDREN, especially adapted to the use of medical students. By Marcus P. Hatfield, A. M., M. D., Professor of Diseases of Children in the Chicago Medical College, etc. With a colored plate. Philadelphia: P. Blakiston, Son & Co.: 1890.

This enterprising publishing company has been getting out a great many excellent little books under the name of "Quiz-Compends." This is No. 14. These books are devoted to special subjects. The present volume has a great deal of practical matter in it. B.

A TEXT-BOOK OF PRACTICAL THERAPEUTICS, with especial reference to the application of remedial measures to disease, and their employment upon rational basis. By Hobart Amory Hare, M. D. (Univ. of Pa.), B. Sc. Pages, 622: Cloth: $3.75: 1890.

This book is practically arranged for the use of physicians and students. It is really a work on therapeutics and practice combined, and it is believed that if a busy physician will familiarize himself with the arrangement, which is indeed simple, he will prefer it to any work now published. An index of the remedies and of the diseases is given in alphabetical order. Special departments are reviewed by specialists, and the whole subject matter brought up to date. B.

---

DISEASES OF THE RECTUM AND ANUS, their Pathology, Diagnosis and Treatment. By Chas. B. Kelsey, A. B., M. D., Professor of Diseases of the Rectum at the New York Post-Graduate Medical School and Hospital, etc. Third edition; rewritten and enlarged, with two chromo-lithographs and one hundred and sixty-eight illustrations: pp. 480: price ——: 1890: New York: Wm. Wood & Co., publishers.

This is a text-book, and stands at the head as authority on the subject of diseases of the rectum and anus. The present edition has been rewritten, and brought up to date in every particular. No one specially interested in the subject can afford to be without Prof. Kelsey's work. It is both scientific and practical, and should be in every physician's library. B.

---

ESSENTIALS OF MINOR SURGERY, Bandaging and Venereal Diseases, arranged in the form of questions and answers. Prepared especially for students of medicine; by Edward Martin, A. M., M. D., Instructor in Operative Surgery, University of Pennsylvania, etc. Illustrated. Philadelphia: W. B. Saunders, 1890. Price, $1.00. Pp. 164.

---

THE PHYSICIAN'S ALL-REQUISITE TIME AND LABOR-SAVING ACCOUNT BOOK, designed by Wm. A. Siebert, M. D., of Easton, Pa. Philadelphia: F. A. Davis, publisher, 1890. No. 1, 300 pages for 900 accounts per year; size 10x12; bound in Russia, $5.00. No. 2, same for 1800 accounts, $8.00.

---

THE MEDICAL STUDENT'S MANUAL OF CHEMISTRY; by R. A. Withaus, A. M., M. D., Professor of Chemistry and Physics in the University of New York, etc. Third edition. New York: Wm. Wood & Co., 1890. Pp. 500.

This work has run through three editions, which evinces its popularity. There is no change in the classification and arrangement of subjects in the present issue. Every advancement has been made in the light of discoveries since the last edition. That portion treating of carbon compounds seems to have been extended farthest. B.

---

EPILEPSY, ITS PATHOLOGY AND TREATMENT; being an essay to which was awarded a prize of four thousand francs by the Academie Royale de Medicine de Belgique, December 31, 1889. By Hobart Amory Hare M. D., (Univ Pa.), B. Sc., Clinical Professor of the Diseases of Children and Demonstrator of Therapeutics in the University of Pennsylvania, etc. Philadelphia and London: F. A. Davis, publisher, 1890. Pp. 222. Price, net $1.25.

This is a prize essay, and to be sure it shows labor, investigation and great care in preparation, still there is nothing specially new in it. Every government should encourage scientific investigations and make annual appropriations of money for such purposes. The subject of epilepsy is ably handled in this little book. B.

---

SAUNDERS' POCKET MEDICAL LEXICON, being a dictionary of words and terms used in medicine and surgery, collated from the highest authorities and brought up to present date. By John M. Keating, M. D. (Univ. of Pa.), and Henry Hamilton. With addenda, consisting of etymological factors common in medical terminalogy; comparative tables of metric and apothecaries weights; a list of poisons and their antidotes, and abbreviations used in prescriptions. Philadelphia. W. B. Saunders, 1890. Pp. 280. Price, 75 cts.

---

ANNUAL OF THE UNIVERSAL MEDICAL SCIENCES. A yearly report of the progress of the general sanitary sciences throughout the world. Edited by Chas. E. Sajous, M. D., and seventy associate editors, assisted by over two hundred corresponding editors, collaborators and correspondents. Illustrated with chromo lithographs, engravings and maps. Five volumes, 1890, F. A. Davis, publisher. Philadelphia, New York, Chicago, Atlanta, London, etc.

This is the third year of publication of this work, and each year decided improvements have been made.

There is still room for improvements in some of the departments. Notably *Diseases of the Rectum and Anus.*

Twelve or thirteen pages are devoted to a controversy on hemorrhoids, in which the editor of the department has been assailed on his advocacy of the clamp and ligature treatment. All of any interest to the public could have been said on a page or two. The same criticism can be made against other departments. The editor is disposed to give too much prominence to his own ideas, and not come up with an impartial review of other important papers of the year.

The chief improvements in these volumes over last year's issue has been the creation of a separate department on Syphilis, by Dr. J. W. White, and on Surgical Mycoses, by Dr. Earnest Laplace, and on Thoracic Surgery, by Dr. J. D. Gaston.

The issue for 1890 is well worth the price asked, and altogether it gives a good resumé of the journal articles for the year. B.

---

OINTMENTS AND OLEATES, Especially in Diseases of the Skin, by John V. Shoemaker, A. M., M. D., Philadelphia. Second edition, revised and enlarged. Philadelphia and London. F. A. Davis, publisher, 1890. Pp. 298. Price $1.50 net.

Part first of this book is devoted to ointments, their therapeutic uses, etc., including all of importance from the U. S. Pharmacopæia, the national formulary. Also official ointments in the British, German, French, Austrian, Italian, Spanish, Mexican and Chilian Pharmacopæias.

Part second is devoted to the oleates, their history, manufacture, therapeutics, etc. The oleates of mercury and zinc have been made officinal in the U. S. P. As Shoemaker demonstrates the value others will be included. B.

---

BACTERIOLOGICAL TECHNOLOGY for Physicians, with seventy-two figures in the text, by Dr. C. J. Solomonsen, authorized translation from the second revised Danish edition, by William Trelease. New York. Wm. Wood & Co., 1890. Pp. 158 Price——

This is a text book and concisely treats of bacteria and bacteriological methods.

Anyone obliged to take up the study at home will find this little volume of great advantage. In fact, he will find by its study that an instructor is not necessary to a clear understanding of the subject. B.

---

A Compend of Equine Anatomy and Physiology; by William R. Ballon, M. M., Professor of Equine Anatomy in New York College of Veterinary Surgeons, etc.; with twenty-nine graphic illustrations, selected from Chauveau's "Comparative Anatomy." Philadelphia: P. Blackiston, Son & Co., 1890. Pp. 205. Price $1.00. Send to publishers.

---

A Compend of Surgery for Students and Physicians; by Greville Horwitz, B. S., M. D., Demonstrator of Anatomy in Jefferson Medical College, etc. Third edition, thoroughly revised, enlarged and improved; with ninety-one illustrations. Philadelphia: P. Blackiston, Son & Co, 1888. Pp. 205. Price $1.00. Send to publishers.

---

Essentials of the Practice of Medicine (double number); arranged in the form of questions and answers, prepared especially for students of medicine; by Henry Morris, M. D., late Demonstrator Jefferson Medical College, etc. With a very complete appendix on the Examination of Urine, by Lawrence Wolff, M. D., Demonstrator of Chemistry Jefferson Medical College. Philadelphia: W. B. Saunders, 1890. Pp. 426. Price $2.50. This book is intended to assist the advanced student in his hour of need—just before his final examination. It is concisely arranged and well bound. B.

---

Essentials of the Diseases of Children; arranged in the form of questions and answers, prepared especially for students of medicine; by William M. Powell, M. D., Physician to the Clinic for the Diseases of Children in the Hospital of the University of Pennsylvania, etc. Philadelphia: W. B. Saunders, 1890. Pp. 214. Price $1.

This little book can be made of considerable service to the student who expects tc come up for graduation and who has but a few hours to run over the subject. This about covers its field of usefulness.

---

POST MORTEMS; What to Look for and How to Make Them; with sections on Infanticide, Poisons, Malformations, etc.; by A. H. Newth, M. D., London. Edited with numerous notes and additions, by F. W. Owen, M. D., Demonstrator of Anatomy in Detroit College of Medicine. Published by the Illustrated Medical Journal Co., Detroit, Mich., 1890. Pp. 134.

